# Senecavirus A Incidence in U.S. Breeding Herds: A Decade of Surveillance Data

**DOI:** 10.3390/ani15111650

**Published:** 2025-06-03

**Authors:** Mariana Kikuti, Xiaomei Yue, Claudio Marcello Melini, Sarah Vadnais, Cesar A. Corzo

**Affiliations:** Department of Veterinary Population Medicine, University of Minnesota, Saint Paul, MN 55108, USA; mkikuti@umn.edu (M.K.); yue00075@umn.edu (X.Y.); melin145@umn.edu (C.M.M.); vadna033@umn.edu (S.V.)

**Keywords:** Senecavirus A, disease surveillance, incidence, epidemiology, swine diseases

## Abstract

Senecavirus A (SVA) is an endemic pathogen with ongoing relevance to the U.S. swine industry due to its clinical resemblance to foot-and-mouth disease (FMD) and its potential impact on animal health and trade. This study analyzes the incidence and geographic distribution of SVA outbreaks in U.S. breeding herds from January 2015 to December 2024. We used enhanced surveillance by combining diagnostic data from veterinary diagnostic laboratories and practitioners’ reports to assess SVA incidence, identifying temporal fluctuations and regional patterns. Our findings indicate that the cumulative incidence of SVA in U.S. breeding herds is low overall (<2.5% per year), with peak incidence observed during the third and fourth quarters of the calendar year. Outbreaks are more frequently reported in the Midwest. Our results suggest potential seasonality or regional factors influencing disease transmission. The median time interval between SVA outbreaks for sites experiencing more than one outbreak is 402 days. This study provides valuable insights into the current epidemiology of SVA in U.S. breeding herds, and highlights the need for continued monitoring and preparedness to prevent future outbreaks.

## Simple Summary

Senecavirus A (SVA) is a virus that affects pigs and causes skin lesions that look similar to those caused by serious foreign animal diseases like foot-and-mouth disease. Because of this, every case requires careful investigation to rule out more severe threats. In 2015, a major outbreak of SVA raised concerns across the U.S. swine industry. However, since then, there has been little research on how often SVA occurs in U.S. pig farms. This study examines how frequently new SVA outbreaks happen in breeding herds to better understand how the virus spreads, as well as when and where it is most commonly detected. Our findings show that while SVA continues to affect U.S. breeding herds each year, it remains relatively uncommon, with only in a small proportion of herds affected. Outbreaks tend to happen more often in the second half of the year, with most cases occurring in the Midwest. Understanding these patterns can help farmers and veterinarians to improve prevention strategies and limit the impact of SVA on swine production.

## 1. Introduction

Senecavirus A (SVA) is a positive-stranded RNA virus belonging to the genus *Senecavirus*, of the family *Picornaviridae* [1]. It was first described in the early 2000s after a few serologically similar viruses were detected in pig specimens across multiple U.S. states over the previous decades [2]. Large-scale outbreaks in swine were first reported in Brazil in 2014 [3], shortly followed by outbreaks in other countries such as China [4], the U.S. [5], Colombia [6], and Thailand [7]. The pathogenesis of SVA infection in sows and finisher pigs involves a short incubation period (3–5 days), followed by variable clinical signs which may include lethargy, lameness, and the formation of vesicles on the snout and feet which rupture and heal within 14–16 days [8,9]. The infection generates a short period of viremia (3–10 days), with virus shedding lasting up to 21–28 days [8]. In neonatal piglets, SVA is associated with sudden death, severe diarrhea, dehydration, and lethargy, which can lead to an increase in pre-weaning mortality rates ranging from 5% to 60%, and diarrhea lasting 1 to 5 days [9].

Between-animal transmission is believed to primarily occur through direct contact with vesicular fluids or lesions, which carry a high viral load, though the virus can also be found in fecal samples [10,11]. Immunohistochemistry of the kidney and urinary bladder shows positive immunoreactivity to SVA, suggesting that urine-contaminated surfaces may also serve as a route of transmission [12,13]. SVA can persist in the tonsils of infected pigs for months, with stress events such as transport or parturition triggering intermittent viremia and shedding [14]. This phenomenon makes within-herd transmission particularly complex in the presence of carrier sows. Moreover, sow-to-piglet transmission can occur both horizontally and, seemingly, vertically, since viremic newborn piglets have been observed in litters from positive sows, and the virus has been detected in colostrum and milk [15].

Studies on SVA introduction to breeding herds suggest that personnel and animal movement, as well as dead animal management, may be significant risk factors for disease transmission [16]. The virus has been isolated from the small intestine and feces of mice from an SVA-positive sow herd, indicating their potential role in within-herd transmission [17]. Similarly, SVA RNA has been detected in houseflies in or around herds experiencing an SVA outbreak, and after exposure to SVA tissue culture [17,18]. Detection of SVA has also been reported in *Culicoides* captured near pigs experimentally infected with SVA, but not in mosquitoes [19]. However, the involvement of these common pests in between-herd transmission, either mechanically or as reservoirs, remains to be investigated, especially since the flight range of houseflies and *Culicoides* is relatively short (averaging 5–7 km for houseflies and a maximum of 4 km for *Culicoides*) [20,21].

Once the clinical signs and vesicular lesions caused by SVA are present, a foreign animal disease (FAD) investigation is needed to differentiate it through laboratory testing from other reportable vesicular diseases of concern (i.e., foot-and-mouth disease, which shares similar vesicular lesions). Since 2016, most foreign animal disease investigations in the U.S. have involved vesicular disease cases, with pigs being the most frequently affected species [22], underscoring the significant burden that diseases like SVA place on state and federal health agencies.

Diagnosis can be performed using serological methods, such as immunofluorescence assay (IFA) to detect IgG antibodies, or molecular methods, like real-time reverse-transcription polymerase chain reaction (rRT-PCR) targeting the SVA 3D polymerase gene in vesicles, blood, tissues, oro-nasal secretions, feces, and environmental samples. Seroprevalence studies have been conducted to assess the global burden of SVA, with an estimated average seroprevalence in the U.S. of 72% based on a meta-analysis comprising studies from 2014 to 2020 [23]. More recently, a 2019 cross-sectional study analyzing samples from 193 pig farms across 17 U.S. states found that the seroprevalence of SVA was higher in breeding farms (17.3%) than in growing farms (7.4%), with breeding farms being 2.64 times more likely to be seropositive [24]. The study also confirmed the widespread presence of SVA, detecting seropositive animals in seven states, including four in the Midwest and three outside of this region [24].

Despite its significance, nationwide current data on SVA occurrence in U.S. pig herds remain limited and not readily available for a comprehensive and ongoing understanding of its national burden. While SVA case counts based on submissions to veterinary diagnostic laboratories (VDLs) are informative, these may include environmental sampling or multiple submissions from the same outbreak, which can potentially lead to overestimation of disease occurrence. Furthermore, since laboratory submissions are likely driven by clinical suspicion, the positivity rate might be higher than expected in the general population. Therefore, we aimed to estimate the cumulative incidence of SVA in breeding herds in the U.S. in order to better characterize the disease burden.

## 2. Materials and Methods

The population studied comprised swine breeding herds participating in the Morrison Swine Health Monitoring Project (MSHMP). The MSHMP is a voluntary initiative led by the University of Minnesota, in which participating production systems enroll their breeding herd sites and report their weekly health status for major endemic diseases such as Porcine Reproductive and Respiratory Syndrome (PRRS) and Porcine Epidemic Diarrhea (PED) [25,26]. Participants also share data from three major VDLs (University of Minnesota, Iowa State University, and South Dakota State University) for PRRS and PED. Currently, the sow population participating in the MSHMP represents approximately 60% of the total U.S. breeding herd. Given its extensive coverage and consistent reporting, the MSHMP provides a robust platform to estimate SVA incidence trends at the national level.

The SVA diagnostic data analyzed in this study were provided by the University of Minnesota (UMN) and Iowa State University (ISU) VDLs from January 2015 to December 2024. Briefly, the data included the type of specimen submitted, the official Premises Identification Number (PIN)—which is a unique, permanently assigned identifier for premises that allow animal health officials to trace animal origins during health or food safety emergencies—and all SVA polymerase chain reaction (PCR) results originating from production systems participating in the MSHMP. In-house reverse-transcription quantitative PCR, based on the National Animal Health Laboratory Network (NAHLN) foot-and-mouth disease (FMD)/SVA duplex assay, was performed at the UMN VDL for SVA diagnosis. Briefly, samples were extracted using a commercial extraction kit (Ambion MagMAX-96 viral RNA isolation kit; Life Technologies, Carlsbad, CA, USA) and tested using the TaqMan Fast Virus 1-Step Master Mix (ThermoFisher, Waltham, MA, USA), with validated primers and probes [27,28], on an ABI 7500 Fast Sequence Detection System (Applied Biosystems, Waltham, MA, USA). Similarly, an in-house real-time PCR assay was performed at the ISU VDL. Samples were extracted using a commercial extraction kit (MagMAX pathogen RNA/DNA kit; Thermofisher, Waltham, MA, USA) and tested using the VetMAX^TM^ Fast Multiplex Master Mix (Thermofisher, Waltham, MA, USA), with confidential primers and probes, on an ABI 7500 fast system (Thermofisher, Waltham, MA, USA). Submissions of environmental samples, isolates, and miscellaneous specimens were excluded from the incidence estimations, as they might not accurately represent clinical cases. Eligible specimens included various tissues and fluids, such as blood, brain tissue, liver tissue, lung tissue, lymph nodes, oral fluids, swabs, vesicles, etc. SVA PCR submissions were linked to MSHMP-monitored sites by their PIN.

To estimate the disease occurrence, we defined the outbreak based on two resources: laboratory surveillance and MSHMP reporting data. Using laboratory surveillance data, an outbreak was defined when a herd had an SVA PCR-positive submission. Positive submissions from the same site within a twelve-week interval were considered to be representative of the same outbreak, and thus not counted as a new case [29]. Using MSHMP reporting data, an outbreak was also considered when a site directly reported an SVA outbreak to the MSHMP despite no positive submission being detected by the laboratory surveillance, as they may have used other VDLs for detection. Laboratory-confirmed and MSHMP-identified outbreaks were deduplicated by retaining the earliest outbreak date when both occurred within twelve weeks. The yearly cumulative breeding herd incidence was calculated using the number of breeding herds reporting weekly statuses to the MSHMP for either PRRS, PED, or SVA (i.e., sites sharing information for at least one of the primary diseases monitored by the MSHMP) as the denominator, and the number of SVA outbreaks as the numerator. The geographical distribution of outbreaks was described based on U.S. Census regions [30].

## 3. Results

**Figure 1:**
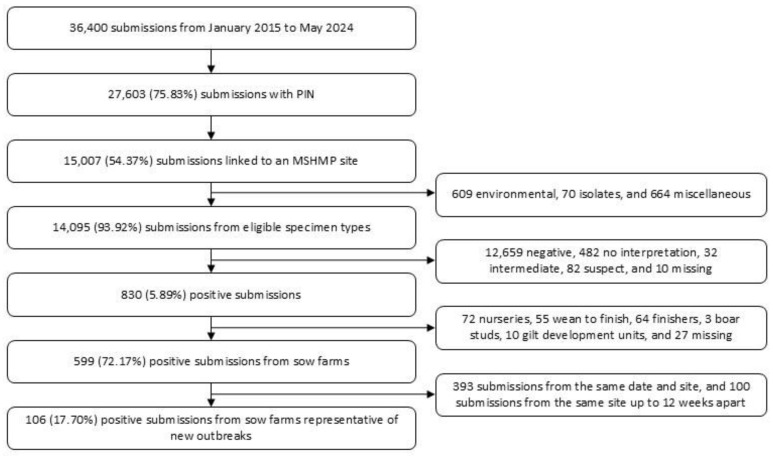
Senecavirus A detection in U.S. breeding herds, monitored by the Morrison Swine Health Monitoring Project, from the University of Minnesota and Iowa State University veterinary diagnostic laboratories between January 2015 and December 2024.

The MSHMP involves a dynamic cohort, so the number of breeding herds monitored throughout the study period varied, ranging between 1063 and 1183 sites per calendar year. Through the laboratory surveillance, a total of 36,400 SVA PCR submissions were obtained. After data cleaning, 106 positive submissions from sow farms representative of new outbreaks were identified via laboratory surveillance (Figure 1). Detailed descriptions of data originating from each of the University of Minnesota and Iowa State University VDLs are available in Figures S1 and S2. Of the 106 SVA outbreaks identified via laboratory surveillance, two were excluded due to the sites not being monitored through the MSHMP at the time the outbreak occurred. An additional 29 SVA outbreaks were reported directly to the MSHMP during the study period. After deduplicating, 114 SVA breeding herd outbreaks were detected in 74 sites from 15 production systems across 12 states.

**Figure 2:**
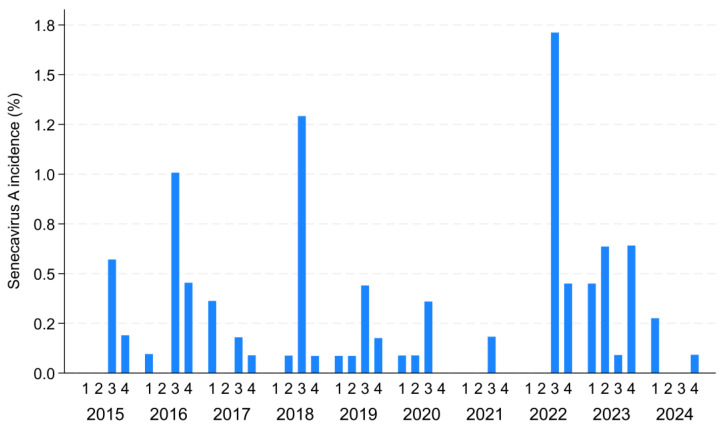
The Senecavirus A (SVA) quarterly cumulative incidence between 2015 and December 2024 among U.S. breeding herds participating in the Morrison Swine Health Monitoring Project.

Most sites experienced only one SVA outbreak (n = 74), while the remaining experienced two (n = 19), three (n = 13), four (n = 5), five (n = 2), and six (n = 1) outbreaks during the study period. For sites that experienced multiple outbreaks, the median time interval between SVA outbreaks was 402 days (interquartile range: 147 days–4 years). Most outbreaks occurred in the third and fourth quarters of the calendar year (65 and 24 outbreaks, respectively), followed by the first and second quarters (15 and 10 outbreaks, respectively), with variations in the quarterly incidence of SVA observed across different years (Figure 2). The majority of outbreaks detected originated in the Midwest (n = 105, 92.11%), followed by the South (n = 8, 7.02%) and the Northeast (n = 1, 0.88%) (Table 1). The yearly cumulative incidence ranged from 0.18% in 2021 to 2.16% in 2022 (Figure S3).

## 4. Discussion

Despite the substantial burden of swine vesicular diseases prompting foreign animal disease investigations in recent years in the U.S., data on the contemporary occurrence of SVA remain limited. Here, we focused on the SVA incidence in breeding herds to leverage ongoing data sharing between producers and academia. The findings suggest that SVA continues to affect U.S. breeding herds, though at an overall low incidence of <2.5%. In the summer of 2015, multiple SVA outbreaks were reported in the U.S. [5,31]. At the time, studies revealed a 1.1% positivity rate in samples submitted to the University of Minnesota and Iowa State University VDLs across all farm types [5,32]. Further screening of Iowa State University submissions from farms with high pre-weaning mortality and diarrhea identified 109 SVA cases by the end of 2015, with cases evenly distributed between breeding and growing pig sites [33]. These findings suggest that the estimated incidence in our study during the heightened concern in 2015 was lower than the positivity rates observed in laboratory-diagnosed cases. This discrepancy may stem from differences in the type of pig populations (i.e., breeding vs. growing) and denominators used for calculation. While incidence estimate disease occurrence within the at-risk population, laboratory positivity rates reflect cases among clinically suspected animals submitted for diagnostic confirmation, which inherently leads to higher positivity rates. Similarly, the positivity rate reported by the Kansas State University veterinary diagnostic laboratory in 2017 was 5.4% [27].

Our study shows a lower SVA disease burden when compared to studies assessing the seroprevalence of SVA. A study screening for clinically negative SVA commercial sow farms, leveraging samples collected in 2016 submitted for PRRSV monitoring at the Iowa State University veterinary diagnostic laboratory, found a within-herd seroprevalence of <20% and a herd-level prevalence of 75.8% [34] using a combination of SVA rVP1 enzyme-linked immunosorbent assay (ELISA) and SVA IgG indirect immunofluorescence assay. More recently, the U.S. SVA herd-level seroprevalence in breeding herds was estimated at 17.3% through SVA IgG IFA. While valuable for assessing past SVA exposure in herds, seroprevalence studies are not well-suited for identifying new infections. This is due to the fact that IgG antibodies have been reported to remain detectable in sows by IFA up to 13 months after an SVA outbreak detection [35]. Thus, they are not directly comparable to our incidence estimations.

Temporal patterns indicate a higher frequency of outbreaks in the third and fourth quarters of the year, suggesting possible seasonal effects. A compilation of reports from multiple VDLs further supports this observation, with a consistent increase in the frequency of SVA cases during summer months [36]. This seasonality requires further investigation, particularly given the limited understanding of between-farm transmission risk factors for SVA. In this study, the SVA incidence was assessed exclusively in breeding herds; however, growing pigs may also play a significant role in transmission, and the disease burden in this population warrants further evaluation. Growing pigs are typically housed in facilities with fewer biosecurity measures than those used for breeding herds, which may influence transmission dynamics. Increased implementation of biosecurity measures has been associated with a protective effect against SVA seropositivity in breeding farms [24]. Additionally, one potential factor contributing to seasonality is vector activity, such as mosquitoes, which exhibit increased activity during periods of higher precipitation [37], and *Culicoides*, with higher population abundance from spring through fall [38]. While their role in SVA transmission requires further investigation, flies and other biting insects are suspected to be primary vectors of other vesicular diseases like vesicular stomatitis in livestock [39].

A steep increase in the estimated SVA incidence was observed in 2022 and 2023. However, the 2024 SVA incidence was the second-lowest, highlighting that the disease burden can change drastically from year to year. Yearly variability in incidence rates may reflect differences in surveillance, reporting, or actual disease fluctuations. The higher incidence in recent years may suggest either an increase in the true prevalence of SVA or improvements in detection and reporting mechanisms. However, despite variations in the yearly number of overall SVA submissions (ranging from 2016 in 2015 to 5586 in 2024; Figure S4), including environmental and other specimens not accounted for in the incidence calculations, no significant changes in SVA surveillance trends over the years were observed.

The fact that most cases occurred in the Midwest highlights this region’s significance for SVA, likely due to it being the region where most of the national pig production is located, which ultimately leads to a high pig density [40]. Nonetheless, this SVA distribution is similar to findings previously reported in 2015, which showed that 96% of the Iowa State University submissions from farms with high pre-weaning mortality and diarrhea were from the Midwest [33]. However, efforts to better assess disease distribution are still needed, particularly since sow herd-level seroprevalence in the U.S. Census South region was found to be higher (33.3%) than in the Midwest (12.3%) [24].

The MSHMP is a voluntary project with a strong focus on PRRS and PED reporting, given the impact that both of these diseases have on production. Participants have been encouraged to report SVA since September 2015, but most cases are still captured through laboratory data, indicating that active reporting of SVA cases through direct communication by the participants remains low. This might have caused some underestimation of SVA cases, particularly early on in the study period. Additionally, the MSHMP was established in 2011 with 13 production companies participating in the project, and has expanded over the years to over 35 production systems which shared retrospective data at enrollment. Thus, under-reporting of older cases by participants might be attributed to recall bias. The MSHMP also does not universally cover the U.S. breeding herd population. Still, because vesicular diseases are included in foreign animal disease investigations, it is likely that we captured the majority of clinical SVA cases when combining participant reporting with VDL surveillance. Some underestimation might also have occurred due to the submission of samples to other VDLs not included in this analysis. However, the University of Minnesota and Iowa State University VDLs conduct the majority of testing among swine production systems in the U.S., likely capturing a significant amount of cases. On the other hand, overestimation might have occurred when considering positive submissions from the same site within 12 weeks as representative of the same outbreak, since farms experiencing an outbreak can test intermittently positive in processing fluids up to 26 weeks after the outbreak [29]. Lastly, this study did not address SVA occurrence in growing pig farms. The herd-level seroprevalence in growing pigs is estimated to be relatively high, ranging from 7.4% to 42.7% [24,34], highlighting this as an important population in SVA epidemiology.

## 5. Conclusions

This study provides current insights into the incidence and distribution of SVA in U.S. breeding herds in the last decade, revealing that while the overall incidence remains low (<2.5%), the virus continues to impact swine populations. The temporal and regional patterns suggest possible seasonal fluctuations and a regional burden, emphasizing the need for continued surveillance to better understand SVA dynamics across the country. Enhanced surveillance systems that integrate on-farm observations and other diagnostic tools could provide a valuable picture of SVA dynamics, which is crucial for developing more effective control measures. Further research should focus on expanding surveillance to include growing pig herds and investigating the potential factors contributing to the observed regional and seasonal variation. Ultimately, this work underscores the importance of collaborative data sharing among producers, veterinarians, and academic institutions to improve the management of SVA and other swine diseases in the U.S., in order to safeguard animal health and ensure the stability of the swine industry.

## Figures and Tables

**Table 1:** Temporal and regional distribution of Senecavirus A outbreaks in U.S. breeding herds participating in the Morrison Swine Health Monitoring Project.

Region	2015	2016	2017	2018	2019	2020	2021	2022	2023	2024	Total
Midwest	7	16	6	17	9	5	2	23	16	4	105
Northeast	0	0	0	0	0	0	0	0	1	0	1
South	1	1	1	0	0	1	0	1	3	0	8

## Data Availability

The data are unavailable publicly due to privacy restrictions, since the data are privately owned by the production companies enrolled in the MSHMP. Data are, however, available upon reasonable request and consent from the production companies involved, though some restrictions might apply.

## Supplementary Materials

The following supporting information can be downloaded at https://www.mdpi.com/article/10.3390/ani15111650/s1: Figure S1. Senecavirus A detection in U.S. breeding herds, monitored by the Morrison Swine Health Monitoring Project, from the University of Minnesota veterinary diagnostic laboratory between January 2015 and December 2024; Figure S2. Senecavirus A detection in U.S. breeding herds, monitored by the Morrison Swine Health Monitoring Project, from the Iowa State University veterinary diagnostic laboratory between January 2015 and December 2024; Figure S3. The Senecavirus A yearly cumulative incidence between 2015 and May 2024 among U.S. breeding herds participating in the Morrison Swine Health Monitoring Project; Figure S4. Senecavirus A yearly submissions to the University of Minnesota and Iowa State University veterinary diagnostic laboratories.

## Abbreviations

The following abbreviations are used in this manuscript:

SVA	Senecavirus A
RNA	Ribonucleic acid
U.S.	United States
FAD	Foreign animal disease
IFA	Immunofluorescence assay
IgG	Immunoglobulin G
rRT-PCR	Real-time reverse-transcription polymerase chain reaction
VDLs	Veterinary diagnostic laboratories
MSHMP	Morrison Swine Health Monitoring Project
PRRS	Porcine Reproductive and Respiratory Syndrome
PED	Porcine Epidemic Diarrhea
PIN	Premises Identification Number
PCR	Polymerase chain reaction
ELISA	Enzyme-linked immunosorbent assay
