# Supplementary figures and images for: Senecavirus A Incidence in U.S. Breeding Herds: A Decade of Surveillance Data
Source: Animals (Basel). 2025 Jun 3;15(11):1650. doi: 10.3390/ani15111650 (PMC12153828; doi:10.3390/ani15111650)

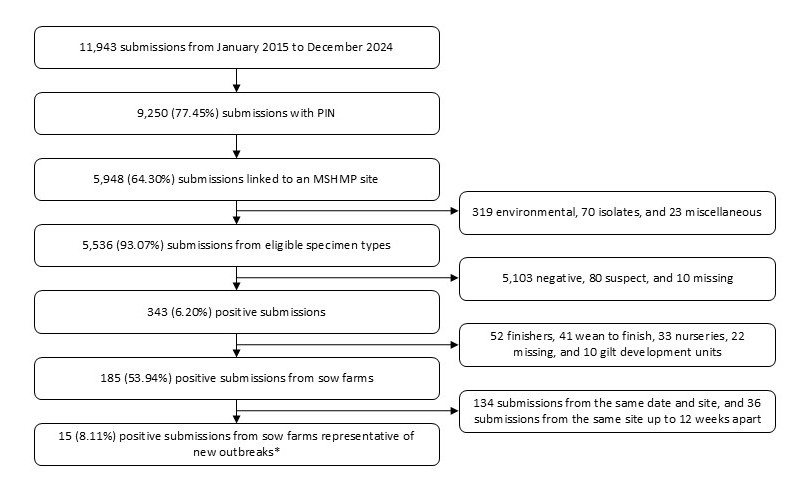

Supplement: Supplementary file 1 [file animals-15-01650-s001.zip › Figure S1.jpg]

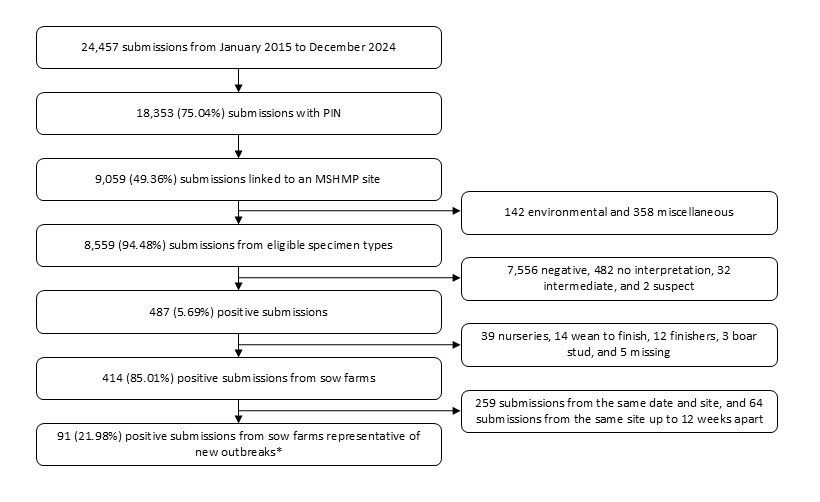

Supplement: Supplementary file 1 [file animals-15-01650-s001.zip › Figure S2.jpg]

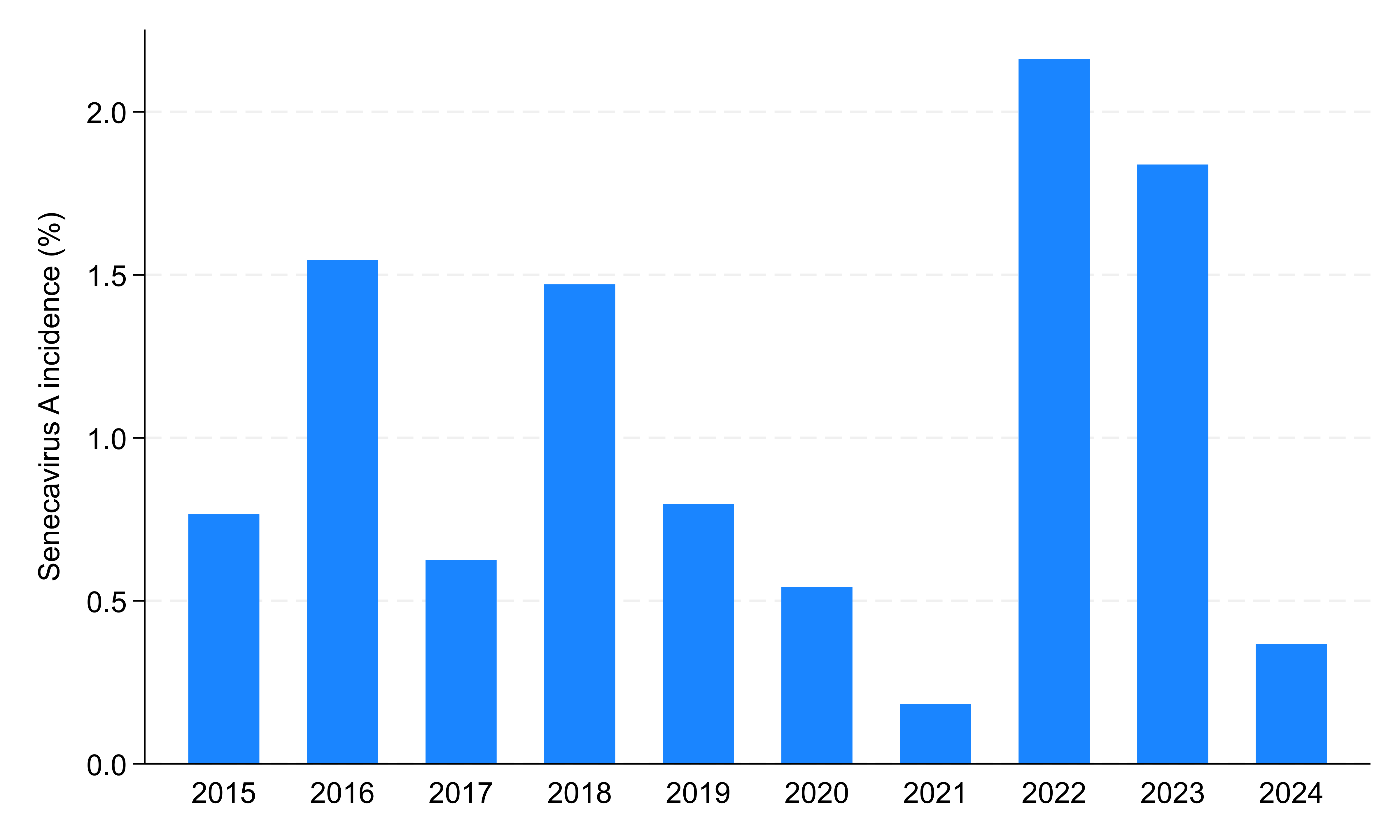

Supplement: Supplementary file 1 [file animals-15-01650-s001.zip › Figure S3.tif]

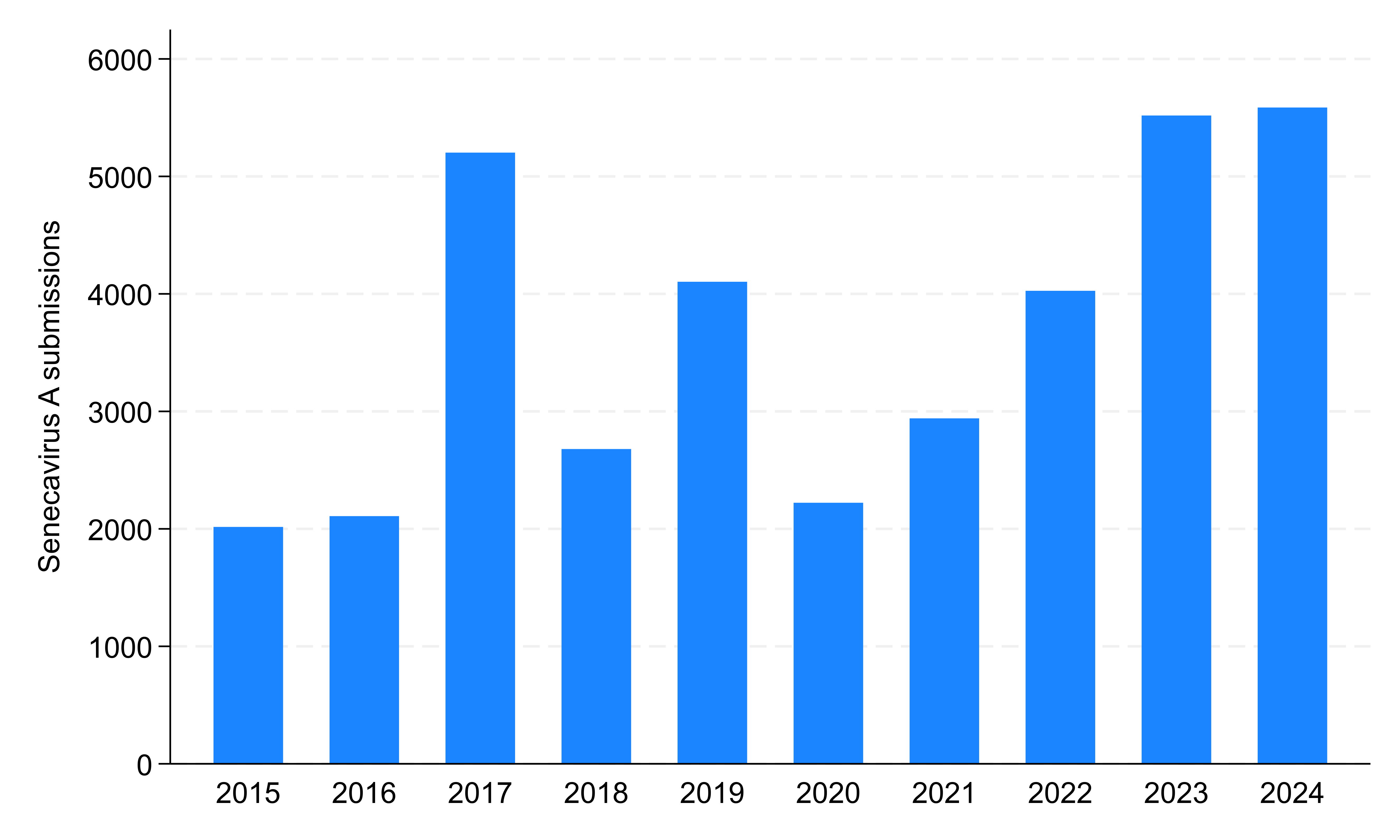

Supplement: Supplementary file 1 [file animals-15-01650-s001.zip › Figure S4.tif]
